# Real-life effectiveness of dupilumab in patients with mild to moderate bronchial asthma comorbid with CRSwNP

**DOI:** 10.1186/s12890-022-02046-3

**Published:** 2022-06-28

**Authors:** Shunsuke Minagawa, Jun Araya, Naoaki Watanabe, Shota Fujimoto, Junko Watanabe, Hiromichi Hara, Takanori Numata, Kazuyoshi Kuwano, Yoshinori Matsuwaki

**Affiliations:** 1 Division of Respiratory Diseases, Department of Internal Medicine, The Jikei University School of Medicine, 3-25-8 Nishi-shinbashi, Minato-ku, Tokyo, 105-8461 Japan; 2 Matsuwaki Clinic Shinagawa, 6-7-29 Kitashinagawa Shinagawa-ku, Tokyo, 140-0001 Japan

**Keywords:** Bronchial asthma, Chronic sinusitis, Dupilumab, Forced oscillation technique, Nasal polyps

## Abstract

**Background:**

Dupilumab, an anti-IL-4α receptor antibody, is a new treatment for severe or refractory asthma. However, real-world evidence on the efficacy of dupilumab in patients with mild to moderate bronchial asthma is lacking.

**Methods:**

We retrospectively evaluated the effects of dupilumab in 62 patients who received dupilumab for eosinophilic sinusitis comorbid with asthma at a single centre in Japan. Type 2 inflammatory markers, ACT, respiratory function tests, and forced oscillation technique (FOT) were analysed before, three months after, and one year after dupilumab administration, mainly in patients with mild to moderate asthma.

**Results:**

FEV1, %FEV1, %FVC, treatment steps for asthma and ACT improved significantly after three months of dupilumab treatment. FeNO was markedly decreased, whereas IgE and eosinophil counts showed no significant changes. Pre- and post-treatment respiratory resistance (Rrs) and respiratory reactance (Xrs) correlated significantly with FEV1. Improvement in %FEV1 was associated with higher FeNO and higher serum IgE before dupilumab treatment.

**Conclusion:**

Dupilumab treatment for sinusitis may improve respiratory functions, asthma symptoms, and asthma treatment reduction, even if the associated bronchial asthma is not severe.

## Introduction

In the last few years, the treatment of refractory asthma has made dramatic progress, especially with the development of biological therapies targeting molecular effectors and signalling associated with type 2 airway inflammation [1–5]. Dupilumab is a fully-humanized IgG4 monoclonal antibody that binds to the IL-4 receptor alpha chain (IL-4Rα) and blocks both IL-4 and IL-13 signalling. Dupilumab has shown efficacy in multiple diseases associated with type 2 inflammation, including asthma, atopic dermatitis, and chronic sinusitis with nasal polyps(CRSwNP) [6]. Several large clinical trials have shown that dupilumab reduces asthma exacerbations, improves the scores on obstructive pulmonary impairment and asthma control tests, and decreases the use of oral corticosteroids(OCS) regardless of peripheral blood eosinophil counts in patients with severe asthma [7–9].

The upper airway, such as the nasal cavity and paranasal sinuses, and the lower airway, such as the bronchi and lungs, are anatomically connected and recognized as the "united airway" [10]. The upper and lower airways are susceptible to functional interactions through hematogenous, transbronchial, and neuromodulatory communication of inflammatory mediators [11–13]. Therefore, it is likely that these two airways are prone to be inflamed simultaneously. It has been reported that 40 to 60% of patients with eosinophilic sinusitis and nasal polyps are complicated with asthma [14, 15]. Tanaka et al. showed that 13 to 20% of eosinophilic sinusitis patients exhibited low FEV1/FVC (< 70%) on respiratory function tests without a previous diagnosis of asthma, indicating that ﻿asthma can be underdiagnosed in patients with CRS. In the SINUS-24 and SINUS-52 trials [16], although the diagnosis of asthma was determined by self-report and patients with severe airflow obstruction were excluded, the asthma complication rate was 60%, further supporting the notion that the actual asthma complication rate may be much higher [15]. Recent reports have shown that nasal polyps and sinusitis treatment may improve asthma symptoms. Ahmed et al. have demonstrated that CRS patients tend to have less overt lower airway disorders, and endoscopic sinus surgery(ESS) effectively ameliorates such disorders [17]. In the pooled population of patients with CRSwNP complicated with asthma from the SINUS-24 and SINUS-52 trials, dupilumab improved forced expiratory volume in one second (FEV1) and patient-reported asthma symptoms evaluated by the Asthma Control Questionnaire (ACQ-6) [16]. This study included all patients regardless of asthma severity. Since dupilumab is approved only for severe or poorly-controlled asthma, no previous reports have analysed the efficacy of dupilumab exclusively in patients with mild to moderate asthma, especially in real-life settings.

The forced oscillation technique (FOT) is a non-invasive, effort-independent method used for respiratory function testing in recent years and is expected to be beneficial, especially in children and the elderly, as it can be performed during tidal breathing [18,19]. However, it is not widely used yet, and its usefulness in asthma treatment has not been fully verified. Furthermore, no previous reports have used FOT to monitor the therapeutic effects of dupilumab in asthma patients.

The present study aims to retrospectively evaluate the real-life effects of dupilumab on comorbid mild to moderate asthma in patients treated with dupilumab for CRSwNP in terms of the changes in type-2 inflammatory markers, ACT, respiratory function tests, and forced oscillation technique (FOT).

## Method

### Study design

This research is a retrospective observational study conducted at a single centre (Department of Otolaryngology and Respiratory Medicine in Matsuwaki Clinic Shinagawa). Sixty-two consecutive adult patients treated with dupilumab for eosinophilic sinusitis with nasal polyps complicated with asthma from May 2020 to July 2021 were retrospectively analysed. All asthma patients were diagnosed by respiratory physicians based on the Japanese guidelines [20] or the Global Initiative of Asthma (GINA) guidelines [21]. All patients received 300 mg of Dupilumab subcutaneously every two weeks. Asthma treatment steps and severity assessment were based on the Global Initiative of Asthma (GINA) strategy updated in 2021 [22]. Blood eosinophil count, IgE, respiratory functions, respiratory impedance, and ACT (Asthma Control Test) were assessed before and at three months and one-year time points after dupilumab administration. A visual analogue scale (VAS) was used to quantify the severity of symptoms of CRSwNP subjectively. The CT findings of CRS were stage-classified using the Lund-Mackay system [23]. The Nasal Polyp Score (NPS) was defined as the sum of the scores (0–8 range) of the right and left nostrils as assessed by nasal endoscopy (a decrease in score indicates improvement). The present study was approved by the Ethics Committee of Jikei university [33–35]. We obtained informed consent in the form of opt-out on the Matsuwaki clinic website, and this study was conducted in accordance with the Declaration of Helsinki.

### Measurement of NO, pulmonary function, and FOT

Fractional exhaled nitric oxide concentration (FeNO) was measured using electrochemical sensor NO analysers (NO Breath, Bedfont Scientific Ltd., England) with a 50 mL/s flow rate, as recommended by the American Thoracic Society / European Respiratory Society, using a spirometer (HI-801, CHEST, Tokyo Japan) that assessed forced expiratory volume in 1 s (FEV1), FEV1/FVC, and (FEV1/forced vital capacity (FVC) ratio). Respiratory impedance was measured by a broadband FOT using MostGraph-02 (Chest MI Co., Ltd, Japan). An impulse oscillatory signal generated by a loudspeaker was applied to the airway through a mouthpiece for about 30 s during tidal breathing.

In this study, we measured and analysed respiratory resistance (Rrs) at 5 Hz (R5) and 20 Hz (R20), and reactance (Xrs) at 5 Hz (X5), resonant frequency (Fres).

### Statistical analysis

Data were analysed using Prism 8 (GraphPad Software Inc., La Jolla, CA, USA). A p-value of < 0.05 was taken as the threshold for statistical significance. ﻿All values are expressed as the means ± standard deviation (SD). Data for individual variables before and three months after starting dupilumab were analysed using the Paired t-test. The factors associated with patient characteristics were analysed using the unpaired t-test and the chi-square test. The Pearson correlation coefficient was used to investigate the correlation between the FEV1 and FOT values.

## Results

### Clinical characteristics

During the observation period, 62 patients underwent respiratory, otolaryngological, and blood examinations after three months of treatment with dupilumab, and 23 patients underwent tests after one year of treatment. During the observation period, all patients who received dupilumab had a past history of asthma or were currently receiving asthma treatment.

All the patients had not experienced any asthma attacks during the past three months and were well controlled by the current asthma treatment (mean ACT score is 22–23) or treatment-free. Of the 62 patients who received dupilumab, 58 (93.5%) received it as their first antibody therapy for asthma, and the remaining 4 (6.5%) had received antibody therapy (omalizumab = 1, mepolizumab = 2, benralizumab = 1) immediately before dupilumab. Most patients in this study received around 5 mg of oral corticosteroids (prednisone) for poorly-controlled sinusitis. Serum IgE and FeNO were mildly elevated than normal in all patients, but there were no significant differences between severe and mild-moderate asthma. The mean serum eosinophil counts were within the normal range regardless of severity. Although there was a mild increase in each of the spirometry indices, as well as the respiratory resistance and reactance in FOT, there were no significant differences in these indices between mild to moderate and severe cases, indicating that asthma treatment was well controlled before the initiation of dupilumab in the present study. The number of patients with mild to moderate asthma was 50, accounting for 80% of all patients, and the efficacy of dupilumab for severe cases has already been established (ref). Accordingly, we performed a series of evaluations mainly focusing on mild to moderate cases (Table 1).

**Table 1:** Baseline patient characteristics are shown

	Mild/moderate	Severe	*p* value
Number of patients(n) (mild/moderate)	50 (21/29)	12	
Observation period(n), 3 month/1 year	50/23	12/0	
Age(y), (sd)	47.1 (9.6)	47.9 (10.6)	0.8225
Female(n),/Male(n)	26/24	6/6	0.9009
Diagnostic history of asthma,n(%)	50 (100)	12 (100)	< 0.0001
Undergoing asthma treatment,n (yes/no)	34/16	12/0 (100)	0.0229
Treatment step (GINA) 1–5,n	2.5	5	< 0.0001
Previous antibody therapy,n(%)	1 (2)	3 (25)	0.0036
Undergoing OCS,n(%)	49 (98)	12 (92)	0.0229
OCSdose(mg), (sd)	4.7 (0.9)	3.8 (2.0)	0.0225
Aspirin intolerance,n(%)	20 (40)	2 (15.3)	0.0972
Asthma control test, (sd)	23.2 (3.0)	22.0 (3.9)	0.2571
IgE, IU/mL (sd)	385 (522)	326 (207)	0.7165
Blood eosinophil count, cells/uL, (sd)	438 (459)	298 (174)	0.3064
FeNO (ppb),(sd)	57.7 (46.6)	75.3 (72.7)	0.302
*Spirometry (pre treatment)*			
FEV1 (L), (sd)	2.9 (0.8)	2.9 (0.8)	0.9074
%FEV1 (%), (sd)	98.5 (16.4)	94.8 (21.1)	0.5192
FEV1%(G) (%), (sd)	81.6 (5.9)	77.8 (8.3)	0.071
%FVC (%), (sd)	106 (18.5)	108 (21.1)	0.9197
*Respiratory impedance (pre treatment)*			
R5 (kPa/(L/s)), (sd)	3.4 (1.0)	3.1 (1.3)	0.4848
R20 (kPa/(L/s)), (sd)	2.9 (1.0)	2.6 (1.1)	0.3052
X5 (kPa/(L/s)), (sd)	-0.4 (0.8)	-0.4 (0.5)	0.9372
Fres (1/s), (sd)	7.8 (3.1)	8.0 (3.2)	0.4194

*p < 0.05, **p < 0.01 by unpaired t test or chi-square test

### Evaluation of clinical parameters in patients with mild to moderate asthma

**Fig. 1:**
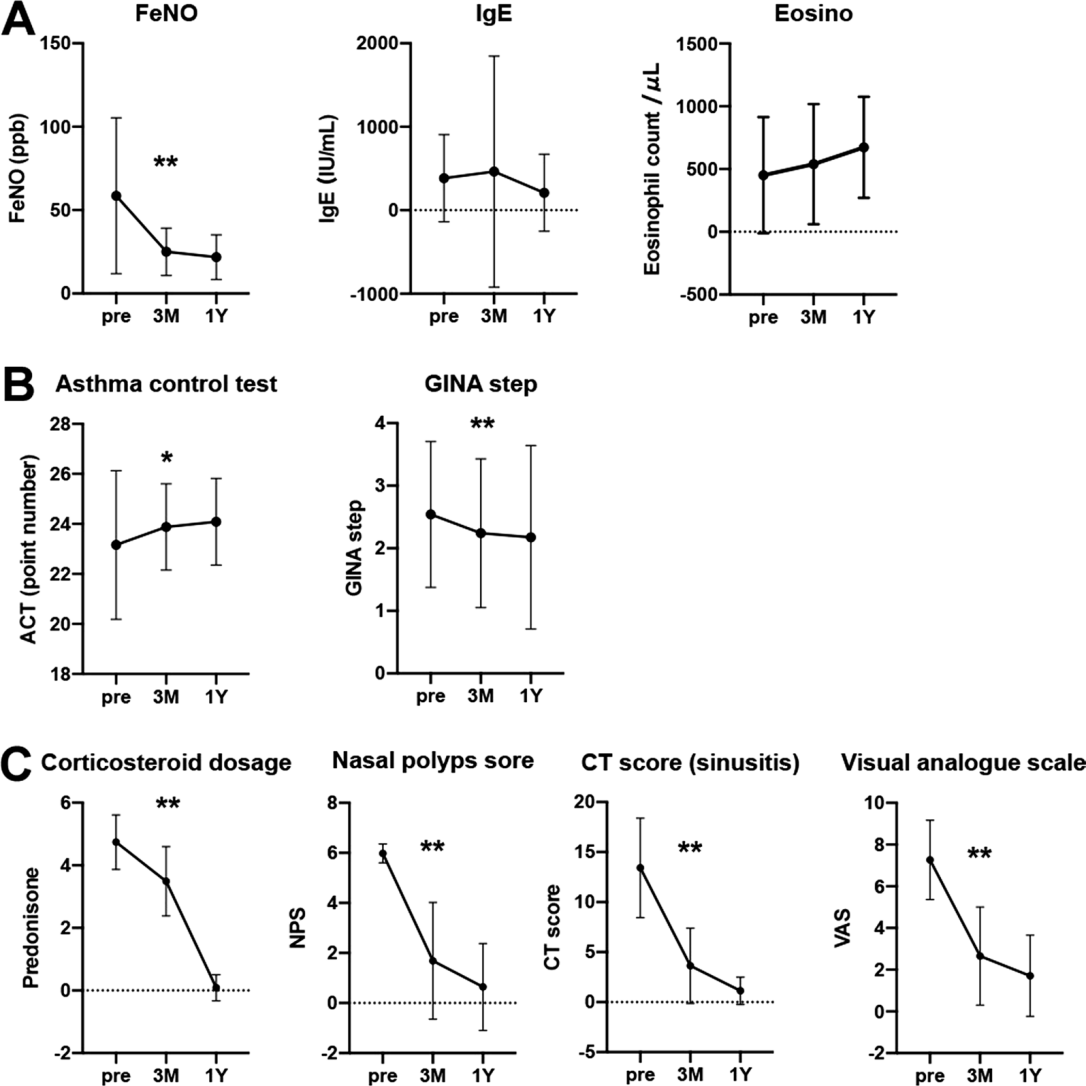
Evaluation of clinical parameters in patients with mild to moderate asthma. Changes in Type 2 inflammatory markers (**A**),asthma control indicators (**B**),and sinusitis treatment Indicators (**C**) at 3 months and 1 year after dupilumab administration are shown in **A**. Pre and 3 month: n = 50, 1 year: n = 18. Each panel shown represents the mean ± SD. *p < 0.05,**p < 0.01 by student’s t test

Figure 1A shows the changes in type 2 inflammatory markers in mild to moderate asthma patients. FeNO decreased markedly at three months after dupilumab treatment and maintained after one year. IgE did not change at three months but showed a decreasing trend at one year (Fig. 1A). The peripheral blood eosinophil counts showed slight elevation without significance until one year (Fig. 1A).

In mild to moderate asthma, the ACT score before treatment was generally good (mean score: 22.9). Nonetheless, three months of dupilumab treatment significantly improved ACT score, which was maintained after one year. In addition, the asthma treatment steps based on the GINA strategy were also significantly downgraded at three months and after one year (Fig. 1B). In terms of CRSwNP-related evaluations, dupilumab dramatically improved the nasal polyp score, CT grade, and odour score (VAS) at three months, which were further improved after one year (Fig. 1C). Furthermore, the steroid therapy for CRSwNP was significantly reduced at the three months, and most patients were successfully withdrawn after one year of treatment with dupilumab (Fig. 1C).

### Assessment of respiratory functions and impedance in patients with mild to moderate asthma

**Fig. 2:**
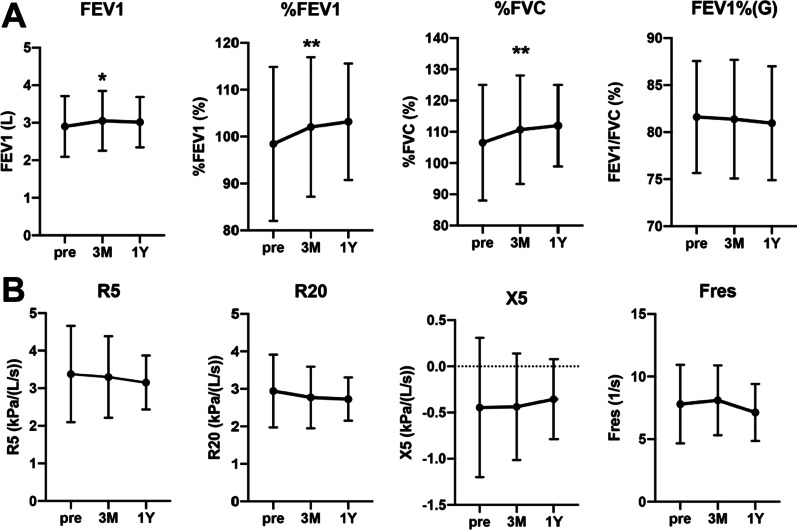
Assessment of respiratory function and impedance in patients with mild to moderate asthma. Changes in respiratory function by spirometry (**A**) and changes in respiratory resistance by FOT (**B**) at 3 months and 1 year after dupilumab administration are shown. Pre and 3 month: n = 50, 1 year: n = 18. Each panel shown represents the mean ± SD. *p < 0.05, **p < 0.01 by student’s t test

With regard to respiratory functions, forced expiratory volume in 1 s (FEV1), the percentage of predicted forced expiratory volume in 1 s (%FEV1), the percentage of predicted forced vital capacity (%FVC), and the FEV1/FVC ratio (FEV1%) were measured by spirometry. The pre-dupilumab values of FEV1, %FEV1, and %FVC were significantly improved at three months, and the improvement was maintained for one year, while FEV1% did not significantly change by dupilumab treatment (Fig. 2A).

As for the respiratory impedance, we measured R5 and R20 as respiratory resistance (Rrs) and X5 and Fres as respiratory reactance (Xrs). Respiratory resistance (R5, R20) tended to improve, but the change was not without significance at three months (Fig. 2B). In addition, all respiratory impedance indices (R5, R20, X5, Fres) showed further improvement after one year, suggesting that one year of dupilumab treatment may significantly improve these indices by accumulating more patients (Fig. 2B).

### Correlation between respiratory function test (FEV1) and respiratory impedance test

**Fig. 3:**
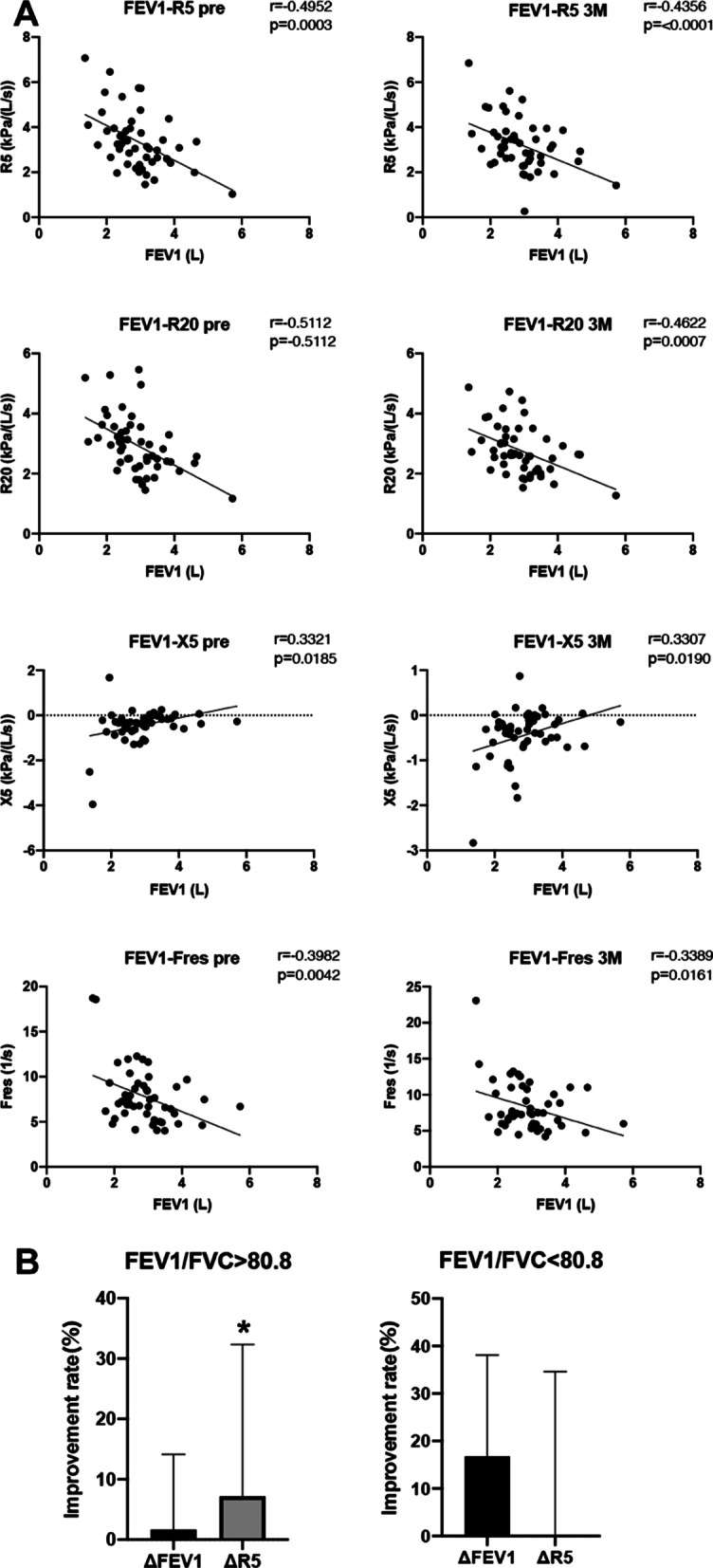
Correlation between respiratory function test (FEV1) and respiratory impedance test. **A** Correlation between respiratory function test (FEV1) and respiratory impedance are shown. n = 50 in each panel. *p < 0.05,**p < 0.01 by the Pearson correlation coefficient. **B** The improvement rates (%) of FEV1 and R5 after three months of dupilumab treatment in patients with FEV1/FVC above and below the mean (80.8%) are shown. n = 50 in each panel. *p < 0.05, **p < 0.01 by unpaired t test

To examine whether FOT is as practical as spirometry in monitoring the effects of dupilumab or not, we evaluated the correlation between FEV1 and respiratory impedance indices (R5, R20, X5, and Fres) before and three months after dupilumab treatment. FEV1 and respiratory impedance correlated significantly before and three months after dupilumab treatment for mild to moderate asthma, suggesting that FOT shows a similar trend to spirometry with respect to obstructive disorders (Fig. 3A). However, when we focused on individual cases, a certain number of cases were found to have discrepancies in the rate of improvement after three months of treatment by spirometry and FOT. Kanda et al. reported that FOT is useful for detecting pathophysiological changes in the respiratory system, even in asthmatic patients with normal FEV1/FVC [24]. Hence, we compared the improvement rates of FEV1 and R5 after three months of dupilumab treatment in patients with FEV1/FVC above and below the mean (80.8%). In patients with above-average FEV1/FVC, the improvement rate of R5 was significantly greater than that of FEV1. Conversely, in patients with FEV1/FVC above average, the improvement rate of FEV1 tended to be larger than that of R5.

### Comparison of the efficacy of dupilumab treatment according to disease severity.

**Fig. 4:**
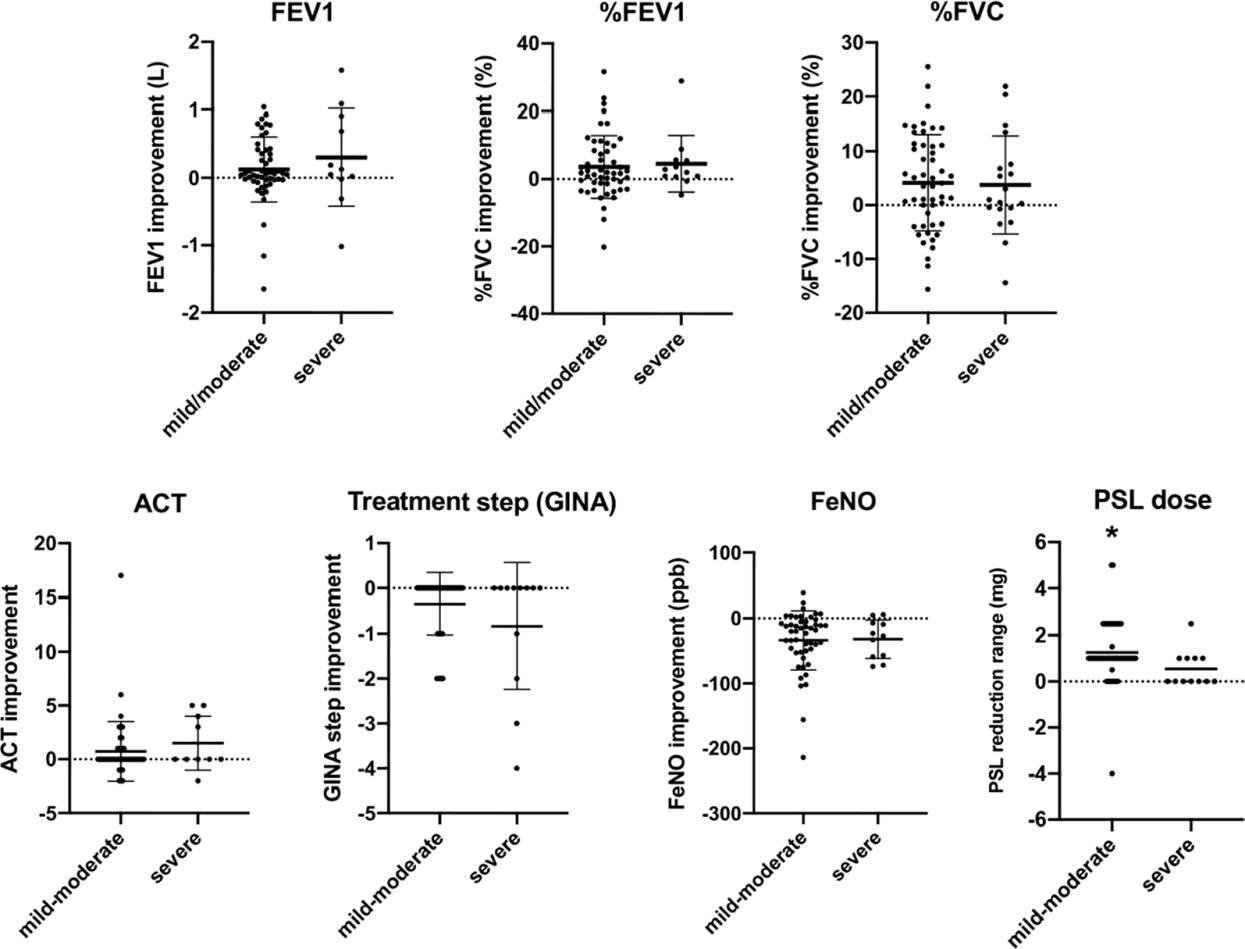
Comparison of the efficacy of dupilumab treatment according to disease severity. The improvement rate of FEV1, %FEV1, %FVC, ACT, treatment-step, and FeNO by asthma severity are shown. Mild-moderate; n = 50, Severe; n = 12. *p < 0.05, **p < 0.01 by unpaired t test

In this study, most patients had mild to moderate asthma, and the efficacy of dupilumab is mainly established in severe asthma. Although all participants, including those with severe symptoms, were clinically controlled at dupilumab administration, we compared the improvement in respiratory functions, ACT, treatment step, and FeNO in response to three months of dupilumab treatment between mild to moderate and severe asthma. The spirometry indices (FEV1, %FEV1, %FVC) showed a trend of improvement in both groups, and no significant difference was observed (Fig. 4). With respect to ACT score, treatment step, and FeNO, mild to moderate asthma, no significant difference was demonstrated between mild to moderate and severe asthma (Fig. 4). Overall, patients with mild-moderate and severe asthma responded similarly to three months of dupilumab treatment in the setting of clinically-controlled cases. Three months of dupilumab treatment reduced the dose of oral corticosteroids (prednisone: PSL) prescribed for refractory sinusitis in most patients, and interestingly, the range of reduction was significantly greater in mild to moderate asthma than in severe asthma.

### Investigation of predictors of improvement in respiratory functions

To determine the patient characteristics contributing to improved respiratory functions with dupilumab in mild to moderate asthma, we compared nine factors associated with asthma and CRSwNP between the group of patients with %FEV improvement below 3.8 L and that with %FEV improvement above 3.8 L. In this study, the reference value was set at 3.8 L, which is the mean improvement in %FEV1 with 3 months of dupilumab treatment for all patients. Among the nine factors, pre-dose FeNO and serum IgE were significantly different between the two groups, suggesting that the higher values of FeNO and IgE can be associated with more improved respiratory functions by dupilumab treatment (Table 2). In addition, a higher treatment step before starting dupilumab contributed to the improvement in %FEV1. The severity of CRS (CT score, VAS, eosinophil cell count in nasal polyps) was not significantly different between the two groups.

**Table 2:** Investigation of predictors of improvement in respiratory function

	A%FEV1 < 3.8% (n = 32)	A%FEV1 > 3.8% (n = 18	*p* value
AERD (no/yes)	19/13	11/7	0.9043
FeNO (ppb),(mean ± SD)	47 (39)	76 (52)	0.0295
IgE, IU/mL (mean ± SD)	232 (198)	625 (753)	0.0133
Blood eosinophil count, cells/uL(mean ± SD)	398 (493)	509 (394)	0.4171
Asthma control test, (mean ± SD)	23.1 (3.5)	23.33 (1.7)	0.7606
Treatment step (GINA),(mean ± SD)	2.3 (1.2)	3.1 (1.1)	0.0336
Preoperative eosinophil count in the nasal polyps(mean ± SD)	153 (231)	128 (46)	0.6703
CT score of sinusitis, (mean ± SD)	12.8 (5.0)	14.6 (4.9)	0.2073
Visual analogue scale 29, (mean ± SD)	7.5 (1.7)	7.3 (2.1)	0.7536

*p < 0.05, **p < 0.01 by unpaired t test or chi-square test

## Discussion

Dupilumab is approved for uncontrolled CRSwNP, severe or uncontrolled bronchial asthma, and atopic dermatitis and has the broadest range of use among antibody products against type 2 inflammation. Although unintentional administration of dupilumab to well-controlled asthma may happen during CRS treatment, respiratory function tests for asthma control are rarely monitored in the clinical practice of otolaryngology. The LIBERTY ASTHMA QUEST trial, a phase-3 trial that showed significant improvement in respiratory functions in bronchial asthma patients with dupilumab, included patients with moderate as well as severe asthma [8, 25]. Similarly, the SINUS trial, a double-blind, randomized, controlled trial of dupilumab in patients with severe CRSwNP, found that 65.9% of patients had moderate asthma at treatment step 3 or 4 in the Global Initiative for Asthma (GINA) guidelines [16]. However, the therapeutic efficacy of dupilumab has not been evaluated to date with a focus on mild to moderate asthma, including asthma in treatment steps 1 and 2. Likewise, no reports have examined the efficacy of dupilumab in well-controlled asthma.

The present study showed that dupilumab significantly improved respiratory functions (FEV1, %FEV1, %FVC) in patients with clinically-controlled mild to moderate asthma associated with CRSwNP at three months of the administration. In addition, asthma treatment steps were significantly reduced after the initiation of dupilumab (Fig. 1B). Patients' subjective symptoms (ACT) were reasonably good before starting dupilumab, but further improvement was demonstrated after three months of treatment (Fig. 1B). Given the marked improvement in each of the CRSwNP-related indices (Fig. 1C), it is plausible that improved upper airway ventilation contributes to the improvement in respiratory symptoms. Actually, there is considerable evidence that surgical treatment of the upper airway can reduce FeNO and serum eosinophil count and improve respiratory functions as well as respiratory symptoms in comorbid asthma [17, 26, 27]. As the potential mechanism, it has been postulated that treatment of CRSwNP affects asthma control by suppressing type-2 inflammation in the upper airway and the whole body. Furthermore, in the QUEST study, patients with concomitant CRS had more remarkable improvements in asthma control than the subgroup without concomitant CRS [28]. Therefore, patients with poorly-controlled CRSwNP in the present study may be a population having susceptibility to dupilumab for asthma treatment even if the comorbid asthma is mild to moderate and clinically controlled.

A posthoc analysis of the LIBERTY ASTHMA QUEST trial showed the beneficial effects of dupilumab (severe exacerbation rates, improved FEV1 and asthma control, and suppressed type 2 inflammatory biomarkers) observed in both allergic and non-allergic asthma patients [25]. However, the therapeutic effect was more significant in patients with elevated type-2 biomarkers (blood eosinophils or FeNO) before dupilumab. In addition, several papers have reported that decreased levels of Th2 inflammatory markers such as FeNO, eotaxin-3, periostin, thymus, and activation-regulated chemokines (TARC), and total IgE can be parameters for monitoring the response to treatment with dupilumab [7–9]. In this study, three months of dupilumab treatment reduced FeNO, the most among Th2 markers, and FeNO also improved %FEV1 in this study (Table 2). These results suggest that FeNO may be an essential type 2 inflammatory marker, which may reflect the susceptibility of asthma associated with CRSwNP against dupilumab treatment.

The forced oscillation technique (FOT) is a respiratory function test that measures respiratory system impedance (Zrs) by applying pressure oscillation of air through the mouth during resting ventilation. The resistance at 5 Hz (R5) reflects the total airway resistance, while that at 20 Hz (R20) reflects the central airway resistance. Respiratory reactance (Xrs) is considered a lung compliance index but can be explained as an index of dynamic obstruction of the peripheral airways, reflecting increased air trapping [29]. Only a few papers on the use of FOT to monitor the effects of the treatment with a biological drug for asthma have been published in the past. Antonicelli et al. reported that FOT parameters showed statistically-significant changes from the third month of mepolizumab treatment in 18 patients with eosinophilic asthma [30]. For the first time, we monitored the effects of dupilumab using FOT in combination with spirometry and evaluated the correlation between the two modalities. R5, R20, X5, and Fres were significantly correlated with FEV1 before and three months after dupilumab administration, suggesting that both modalities can be useful for monitoring efficacy (Fig. 3). However, all indices of FOT showed a tendency of improvement from the third month without statistical significance (Fig. 2B). Higaki et al. suggest that the long-term duration of asthma is associated with a reduction in the reversibility of airway resistance (R20) with bronchodilators in patients with mild to moderate asthma [31]. Thus, we need to evaluate FOT in conjunction with spirometry because we may underestimate the value of FOT in patients with a long duration of asthma. FOT has been considered sensitive in detecting peripheral airway obstruction in asthma [32, 33]. Hsiao et al. reported that asthmatic patients with respiratory symptoms who have normal spirometry may have small airway dysfunctions (SAD) and that IOS (especially reactance) can identify SAD more sensitively than spirometry [34]. Furthermore, in the present study, the improvement rate of R5 after three months of treatment with dupilumab in patients with good FEV1/FVC was significantly higher than that of FEV1. Conversely, the improvement rate of FEV1 in patients with low FEV1/FVC tended to be higher than that of R5. In summary, because of the difference in detection sensitivity between spirometry and FOT depending on the asthma duration and the respiratory functions, it is desirable to perform a combined assessment of spirometry and FOT for treatment evaluation.

There are several limitations to this study. Because of the retrospective observational study, the present study could not be compared with the placebo control. In addition, although this study evaluated 50 patients primarily for the treatment effectiveness over three months, there were still 23 patients who were evaluated for testing after one year. The TRAVERSE study, which followed patients with asthma who had completed a clinical trial of dupilumab for an extended period, showed that the safety and efficacy of dupilumab in patients with severe asthma were maintained even after extending the treatment to 148 weeks [35]. In the present study involving mild to moderate asthma associated with CSRwP, it will be essential to follow the outcome in respiratory functions and symptoms for a more extended period.

This study is unique in that it examined the response of patients with well-controlled asthma to treatment, unlike previous studies that examined the effects of treatment with dupilumab in severe asthma patients. This study is also unique in that all patients had sinusitis that was poorly controlled after sinusitis surgery alone, which is not possible in the usual practice of respiratory medicine alone. In summary, dupilumab therapy not only has a marked effect on CRSwNP but may also improve respiratory functions and symptoms and reduce the dose of asthma treatment, even if comorbid asthma is not severe and is clinically controlled.

## Data Availability

The datasets generated and/or analysed during the current study are not publicly available due to confidentiality issues and to safeguard accurate data interpretation, but are available from the corresponding author on reasonable request.

## References

[1] Numata T, Miyagawa H, Nishioka S, Okuda K, Utsumi H, Hashimoto M, et al. Efficacy of benralizumab for patients with severe eosinophilic asthma: A retrospective, real-life study. BMC Pulm Med. 2020;20(1). 10.1186/s12890-020-01248-x PMC7398222 32746787

[2] Numata T, Araya J, Miyagawa H, Okuda K, Fujita Y, Utsumi H (2021). Effectiveness of switching biologics for severe asthma patients in Japan: a single-center retrospective study. J Asthma Allergy.

[3] Harada N, Ito J, Takahashi K (2021). Clinical effects and immune modulation of biologics in asthma. Respir Investig.

[4] Napoletano F, Baron O, Vandenabeele P, Mollereau B, Fanto M (2018). Intersections between regulated cell death and autophagy. Trends Cell Biol.

[5] Dragonieri S, Carpagnano GE. Biological therapy for severe asthma. Asthma Res Pract. 2021;7(1). 10.1186/s40733-021-00078-w PMC8362167 34389053

[6] Peters MC, Wenzel SE (2020). Intersection of biology and therapeutics: type 2 targeted therapeutics for adult asthma. Lancet.

[7] Wenzel S, Castro M, Corren J, Maspero J, Wang L, Zhang B (2016). Dupilumab efficacy and safety in adults with uncontrolled persistent asthma despite use of medium-to-high-dose inhaled corticosteroids plus a long-acting β2 agonist: a randomised double-blind placebo-controlled pivotal phase 2b dose-ranging trial. Lancet.

[8] Castro M, Corren J, Pavord ID, Maspero J, Wenzel S, Rabe KF (2018). Dupilumab efficacy and safety in moderate-to-severe uncontrolled asthma. N Engl J Med.

[9] Rabe KF, Nair P, Brusselle G, Maspero JF, Castro M, Sher L (2018). Efficacy and safety of dupilumab in glucocorticoid-dependent severe asthma. N Engl J Med.

[10] Kanda A, Kobayashi Y, Asako M, Tomoda K, Kawauchi H, Iwai H (2019). Regulation of interaction between the upper and lower airways in united airway disease. Med Sci.

[11] Hens G, Raap U, Vanoirbeek J, Meyts I, Callebaut I, Verbinnen B (2011). Selective nasal allergen provocation induces substance P-mediated bronchial hyperresponsiveness. Am J Respir Cell Mol Biol.

[12] Braunstahl GJ, Overbeek SE, KleinJan A, Prins JB, Hoogsteden HC, Fokkens WJ (2001). Nasal allergen provocation induces adhesion molecule expression and tissue eosinophilia in upper and lower airways. J Allergy Clin Immunol.

[13] Higashi N, Taniguchi M, Mita H, Kawagishi Y, Ishii T, Higashi A (2004). Clinical features of asthmatic patients with increased urinary leukotriene E4 excretion (hyperleukotrienuria): Involvement of chronic hyperplastic rhinosinusitis with nasal polyposis. J Allergy Clin Immunol.

[14] John Staniorski C, Price CPE, Weibman AR, Welch KC, Conley DB, Shintani-Smith S (2018). Asthma onset pattern and patient outcomes in a chronic rhinosinusitis population. Int Forum Allergy Rhinol.

[15] Laidlaw TM, Bachert C, Amin N, Desrosiers M, Hellings PW, Mullol J (2021). Dupilumab improves upper and lower airway disease control in chronic rhinosinusitis with nasal polyps and asthma. Ann Allergy, Asthma Immunol..

[16] Bachert C, Han JK, Desrosiers M, Hellings PW, Amin N, Lee SE (2019). Efficacy and safety of dupilumab in patients with severe chronic rhinosinusitis with nasal polyps (LIBERTY NP SINUS-24 and LIBERTY NP SINUS-52): results from two multicentre, randomised, double-blind, placebo-controlled, parallel-group phase 3 trials. Lancet..

[17] Youssef AM, Abdel-Naby Awad OG, Taha M. Pulmonary function of patients with chronic rhinosinusitis and the impact of endoscopic sinus surgery. http://oto-open.org 10.1177/2473974X17738759 PMC6239150 30480195

[18] Cavalcanti JV, Lopes AJ, Jansen JM, Melo PL (2006). Detection of changes in respiratory mechanics due to increasing degrees of airway obstruction in asthma by the forced oscillation technique. Respir Med.

[19] Ohishi J, Kurosawa H (2011). Time lag between oscillatory pressure and flow affecting accuracy of forced oscillation technique. Biomed Eng Online.

[20] Ichinose M, Sugiura H, Nagase H, Yamaguchi M, Inoue H, Sagara H (2017). Japanese guidelines for adult asthma 2017. Allergol Int.

[21] Global initiative for asthma: Asthma management and prevention, 2019. 2019;49(5).

[22] GINA. Global strategy for asthma management and prevention (2021 update). 2021;

[23] Lund VJMI (1993). Staging in rhinosinusitus. Rhinology.

[24] Kanda S, Fujimoto K, Komatsu Y, Yasuo M, Hanaoka M, Kubo K (2010). Evaluation of respiratory impedance in asthma and COPD by an impulse oscillation system. Intern Med.

[25] Corren J, Castro M, O’Riordan T, Hanania NA, Pavord ID, Quirce S (2020). Dupilumab efficacy in patients with uncontrolled, moderate-to-severe allergic asthma. J Allergy Clin Immunol Pract..

[26] Hamada K, Oishi K, Chikumoto A, Murakawa K, Ohteru Y, Matsuda K (2021). Impact of sinus surgery on type 2 airway and systemic inflammation in asthma. J Asthma..

[27] Soler ZM, Jones R, Le P, Rudmik L, Mattos JL, Nguyen SA (2018). Sino-Nasal outcome test-22 outcomes after sinus surgery: a systematic review and meta-analysis. Laryngoscope.

[28] Maspero JF, Katelaris CH, Busse WW, Castro M, Corren J, Chipps BE (2020). Dupilumab efficacy in uncontrolled, moderate-to-severe asthma with self-reported chronic rhinosinusitis. J Allergy Clin Immunol Pract..

[29] Kelly VJ, Sands SA, Harris RS, Venegas JG, Brown NJ, Stuart-Andrews CR (2013). Respiratory system reactance is an independent determinant of asthma control. J Appl Physiol.

[30] Antonicelli L, Tontini C, Marchionni A, Lucchetti B, Garritani MS, Bilò MB (2020). Forced oscillation technique as method to document and monitor the efficacy of mepolizumab in treating severe eosinophilic asthma. Allergy Eur J Allergy Clin Immunol.

[31] Higaki N, Iwamoto H, Yamaguchi K, Sakamoto S, Horimasu Y, Masuda T (2021). Correlations of forced oscillometric bronchodilator response with airway inflammation and disease duration in asthma. Clin Respir J.

[32] Crisafulli E, Pisi R, Aiello M, Vigna M, Tzani P, Torres A (2016). Prevalence of small-airway dysfunction among COPD patients with different GOLD stages and its role in the impact of disease. Respiration.

[33] Usmani OS, Singh D, Spinola M, Bizzi A, Barnes PJ (2016). The prevalence of small airways disease in adult asthma: a systematic literature review. Respir Med.

[34] Hsiao YH, Su KC, Lee YC, Ko HK, Perng DW (2020). Small airway dysfunction by impulse oscillometry in symptomatic patients with preserved pulmonary function. J Allergy Clin Immunol Pract.

[35] Wechsler ME, Ford LB, Maspero JF, Pavord ID, Papi A, Bourdin A, et al. Long-term safety and efficacy of dupilumab in patients with moderate-to-severe asthma (TRAVERSE): an open-label extension study. Lancet Respir Med. 2021; https://www.sciencedirect.com/science/article/pii/S2213260021003222 10.1016/S2213-2600(21)00322-2 34597534

